# Low Measles Seropositivity Rate among Thai Adolescents in the Thai National Immunization Program

**DOI:** 10.3390/vaccines10081269

**Published:** 2022-08-06

**Authors:** Thanyawee Puthanakit, Suvaporn Anugulruengkitt, Piyada Angsuwatcharakon, Pornumpa Bunjoungmanee, Ekasit Kowitdamrong, Athiwat Primsirikunawut, Sukkrawan Intarakhao, Panadda Chetsonwisorn, Jiratchaya Sophonphan, Auchara Tangsathapornpong

**Affiliations:** 1 Division of Infectious Diseases, Department of Pediatrics, Faculty of Medicine, Chulalongkorn University, Bangkok 10330, Thailand; 2 Center of Excellence for Pediatric Infectious Diseases and Vaccines, Chulalongkorn University, Bangkok 10330, Thailand; 3 Department of Diseases Control, Ministry of Public Health, Nonthaburi 11000, Thailand; 4 Division of Infectious Diseases, Department of Pediatrics, Faculty of Medicine, Thammasat University, Pathumthani 12120, Thailand; 5 Division of Virology, Department of Microbiology, Faculty of Medicine, Chulalongkorn University, Bangkok 10330, Thailand; 6 National Institute of Thailand, Department of Medical Sciences, Ministry of Public Health, Nonthaburi 11000, Thailand

**Keywords:** seroprevalence, measles, children, adolescents, Thai

## Abstract

To achieve the goal of measles elimination, herd immunity with 95% seroprotection in the community is required. This study aimed to describe the measles seropositivity rate among Thai children and adolescents. A cross-sectional study was conducted among children aged 3–18 years in Bangkok and its suburbs. Measles IgG antibodies were measured using a EUROIMMUN enzyme-linked immunosorbent assay kit. Seropositivity is defined as a measles IgG titer of ≥200 IU/L, due to a correlation with a >85% positive rate with a plaque reduction neutralizing titer of >120. Factors associated with seropositivity were analyzed using logistic regression analysis. From May to July 2020, 570 children with a median (IQR) age of 11.7 (9.4–14.8) years were enrolled. The geometric mean titer (GMT) of anti-measles IgG was 281 IU/L (95% CI; 257–306). The proportion of children with seropositivity was inversely correlated with age; 3–5 years 85.3%, 6–9 years 72.5%, 10–14 years 50.7%, and 15–18 years 56.3%. Adolescents aged 10–18 years had a lower measles seropositivity rate compared with young children; aOR 0.29 (95% CI 0.17–0.48). Only half of the adolescents who received two doses of measles-containing vaccine maintained measles IgG above the seropositive level. A measles booster dose for young adults may be needed to achieve the measles elimination goal.

## 1. Introduction

Measles is a viral respiratory illness with an airborne mode of transmission, making it highly contagious. Before the introduction of the measles vaccine in 1963, measles caused an estimated 30 million cases globally, with more than 2 million deaths per year [1]. Complications associated with measles infection include pneumonia, and a chronic neurological condition known as subacute sclerosing panencephalitis (SSPE). The risk of complications is increased among infants, adults older than 20 years of age, and persons with immune suppression [2,3].

Since 1984, the Thai National Immunization Program (NIP) has recommended vaccinating infants aged 9–12 months against measles. In 1997, the NIP program started recommending a two-dose regimen of the measles vaccine. Subsequently in 2010, the measles vaccine was replaced by a vaccine providing protection against three viruses at once, called the measles-mumps-rubella (MMR) vaccine. The first dose of MMR is given to infants between the ages of 9–12 months, and a booster dose is given at 4–6 years of age. Later in 2014, the booster dose age was shifted from 4–6 years of age to 2.5 years [4]. The coverage of measles immunization in Thailand was >99% for the first dose and 91–95% for the booster dose since 2013 [4,5]. From 2014 to 2016, the overall incidence of measles in Thailand remained as low as 1.8–2.8 cases per 100,000 of the population. However, there was a re-emergence of measles, with the number of reported cases increasing to 10.0 cases per 100,000 of the population in 2018–2019 [6]. This might be explained by several factors, such as primary vaccine failure due to the administration of the first dose of the measles vaccine to infants before the age of 12 months, antibody levels from the vaccine waning in the young adult population due to a long period without exposure, or vaccine breakthrough due to mutant strains in different clades such as B3, and D8 [7,8]. The re-emergence of measles is a phenomenon that is occurring worldwide, with the highest reported cases in 2018 of 140,000 measles deaths globally [9,10].

There are various tools to assess the immunity of the population, such as immunization coverage, disease surveillance, and serosurveillance. Serosurveys demonstrate the presence of antibodies and provide information on population immunity. It is simple lab assay which allows hundreds or thousands of participants to enroll, and characterizes immunity at the population level. However, it may underestimate the benefits of the vaccine, since it does not include the components of cellular-mediated immunity, or neutralizing antibodies, which correlate more with vaccine efficacy. In order to achieve the measles elimination goal, measles immunity levels in the population should be as high as 89–95% [11,12]. A recent analytic study showed that age-specific targets for immunity levels (of 85% in under-5-year-olds, 90% in 5 to 9-year-olds, and 95% in all older age groups) would be necessary to achieve and maintain measles elimination [13]. The plaque reduction neutralization test (PRNT) is regarded as the gold standard of measuring measles antibodies because it is a functional antibody assay. However, PRNT has limitations, such as being labor-intensive, difficult to standardize between different laboratories, and requiring the Edmonston wild-type strain. Measuring measles IgG antibody levels using the ELISA method is common in serosurveillance studies because the ELISA method is simple and automated, but the sensitivity is in the range of 78–93%, which is less than the PRNT method [14,15]. The main difference is that PRNT measures neutralizing antibodies mainly against the measles virus hemagglutinin protein (H protein), whereas the ELISA measures antibodies to the measles nucleocapsid (N protein) [14,15].

Seroprevalence surveys of measles IgG among children and adolescents in Thailand have been conducted every decade. The study conducted in 2004 reported a trend of lower seroprotective rates among children aged less than 15 years compared with young adults (73–76% versus 82% among young adults aged 15–19 years) [16]. A more recent seroprevalence Thai study in 2014 reported a similar measles seroprotective rate of 73.2–74.1% in adolescents and young adults younger than 25 years of age, and 98.7% in adults older than 30 years of age [17]. The objective of this study is to describe the current measles seropositivity among children and adolescents who lived in the Bangkok metropolitan area in 2020, since there were changes in the national recommendation in the past decade, with lower ages at the time of measles’ primary vaccination and booster doses. This approach shows the potential risk of being more susceptible to measles at the population level during the adolescent and young adult period.

## 2. Materials and Methods

### 2.1. Study Population

This study is a cross-sectional seroprevalence study, combining data from two research protocols to cover healthy children from 3 to 18 years of age. The first protocol, a cross-sectional study among healthy adolescents aged 10–18 years (*n* = 424), enrolled participants from May to July 2020 from two clinical research sites: the Faculty of Medicine, Chulalongkorn University, and the Faculty of Medicine, Thammasat University, Pathum Thani, Thailand. The exclusion criteria were: having a chronic illness(es); receiving blood products within the previous 4 weeks; or having a history of receiving measles-containing vaccines (either the measles vaccine or the measles-mumps-rubella (MMR) vaccines) after the age of seven. This study was approved by the Institutional Review Board Faculty of Medicine, Chulalongkorn University (IRB no 686/62) and the Faculty of Medicine, Thammasat University (IRB no 069/2563). The second protocol study participants were healthy children aged 3–9 years (*n* = 146) who had previously had their blood samples collected and stored during 2018–2019 as a baseline sample when they participated in another vaccine study at the Faculty of Medicine, Chulalongkorn University (IRB no. 609/62). Demographic data and immunization records were available without the participant’s personal identification.

### 2.2. Study Procedures

All participants and their parents signed assent and written consent forms as appropriate prior to enrollment. Data on the participant’s history of measles-containing vaccines was collected by reviewing their personal immunization booklet, or taking a history from the participant’s parents in combination with information from the national immunization program vaccine schedule for children in each birth cohort. The Thai NIP used the measles vaccine Edmonston strain, which has a higher immunogenicity response compared with the Schwarz or Moraten strain. For children in the same birth cohort born from 2002 to 2012, the Thai NIP recommended that they received two doses of the measles vaccine: the first dose between the ages of 9–12 months and the second dose was given at 4–6 years of age. Children born from 2012 to 2016 were recommended to receive two doses of the MMR vaccine: at 9–12 months, and at 2.5 years of age. A single blood draw was performed to collect serum for measles antibody assays, of which aliquots were kept at −70 °C until testing.

### 2.3. Measles Antibody Assays

Measles-specific IgG was measured using an Anti-Measles Virus ELISA (IgG) kit (EUROIMMUN, Lübeck, Germany) on a fully automated EUROIMMUN Analyzer I-2P (EUROIMMUN, Lübeck, Germany) according to the manufacturer’s instructions [18]. Laboratory assays were performed at the Department of Microbiology, Faculty of Medicine, Chulalongkorn University. Measles-specific IgG was reported as International Units per liter (IU/L). Measles neutralizing antibody by plaque reduction neutralization test (PRNT) [14] was performed at the Department of Medical Science, Ministry of Public Health in order to define the measles IgG cut-off for this study. In brief, Vero cell monolayers were infected with a low-passage Edmonston measles virus strain and incubated with serially diluted serum specimens in duplicate. The 50% endpoint titers were interpolated using the Karber method [15]. We randomly selected 44 serum samples from participants with different measles IgG levels, then calculated the proportion of serum which met the cut-off criteria of PRNT > 120 mIU. There was 100% correlation if the level of measles IgG antibody was >275 IU/L (*n* =12), followed by 85.7% if the measles IgG titer was between 200 and 275 IU/L (*n* = 14), and 72.2% if the measles IgG titer was between 120 and <200 IU/L (*n* = 18). Measles seropositivity in this study was defined as a measles IgG titer of >200 mIU; the measles IgG cut-off was defined as >120 IU/L (low titer cut-off), and >275 IU/L (high titer cut-off) was used as the sensitivity analysis of measles seroprotective levels.

### 2.4. Statistical Analysis

The geometric mean titer (GMT) of measles IgG was presented with a 95% confidence interval (95% CI) stratified by age group and vaccination status. Differences in GMT between the two groups were assessed using two independent *t*-tests and an analysis of variance (ANOVA) was conducted when comparing the three groups. The seropositivity rate was reported using a measles IgG level of >200 mIU/mL according to the previous report [13]. We also performed sensitivity analysis using different cut-offs, such as a measles IgG level of >120 mIU/mL or >200 mIU/mL or >275 mIU/mL to address the potential waning of measles IgG antibodies and the lower sensitivity of measles IgG tests compared to the PRNT assay. A chi-square test was used to compare SPR rates among age groups. Univariate and multivariate logistic regression analyses were performed to determine the factors associated with the seropositivity rate. Multivariate models were developed by adjusting for covariates with *p* < 0.1 in the univariate model. Statistical significance was defined as *p* < 0.05. Stata version 15.1 (Stata Corp., College Station, TX, USA) was used for analysis.

## 3. Results

The study included data from 570 participants; 422 adolescents aged 10–18 years and 148 children aged 3–9 years in Thailand. Demographic data and history of vaccination are shown in Table 1. Overall median (IQR) age was 11.7 (9.4–14.8) years. About two-thirds of participants (396 participants, 69.5%) had personal immunization booklets available for review. The median age of participants with unavailable immunization booklets was 14.3 years, which was older than participants with documented MCV (*p* < 0.001). The median time interval from the latest measles-containing vaccine to blood draw was 3.0 (2.3–3.5) years among children 3–9 years of age and 7.0 (5–9.4) years among adolescents 10–18 years of age (*p* < 0.001), respectively.

### 3.1. Measles Seropositivity Rate

**Figure 1:**
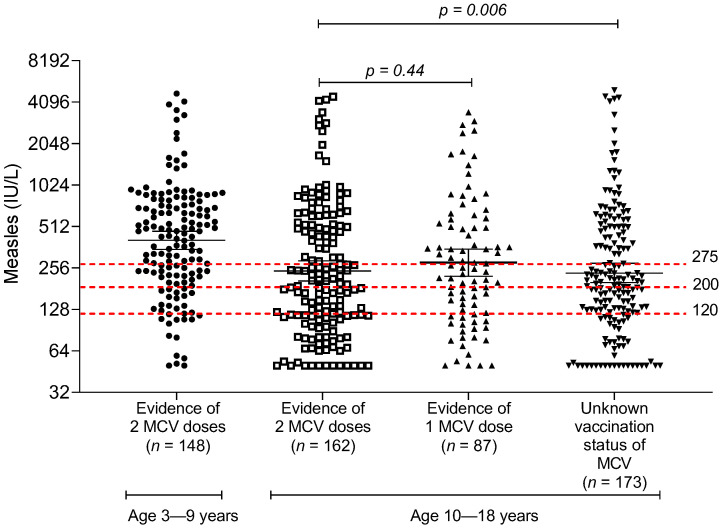
Measles IgG antibody measured by ELISA method, stratified by age group and documentation of the number of measles vaccine doses in the immunization booklet; MCV, measles-containing vaccine.

The distribution of measles IgG titer among children and adolescents is shown in Figure 1. The overall GMT of anti-measles IgG was 281 IU/L (95% confidence interval (CI) 257–306). There was a trend of higher anti-measles IgG among young children aged less than 10 years, compared with children and adolescents aged 10 years and older.

Overall, the measles seropositivity rate using measles IgG > 200 IU/L as the cut-off criterion was 59.1% (95% CI 55.0–63.2). The proportion of children with seropositivity was inversely correlated with the age group; age 3–5 years 85.3%, age 6–9 years 72.5%, age 10–14 years 50.7%, and 15–18 years 56.3%, respectively (Table 2). A sensitivity analysis of the measles seropositivity rate among children and adolescents using low and high measles IgG cut-offs is shown in Table 3. With a high measles IgG titer cut-off (measles IgG level of >275 IU/L), only 46.3% (95% CI 42.2–50.5) of children in the cohort met the criteria for seropositivity. Even young children 3–5 years of age who just recently received two doses of the MMR vaccine had only 72.1% seropositivity. With the low measles IgG titer cut-off (measles IgG level of >120 IU/L), the overall seropositivity was 77.2% (95% CI 73.5–80.6), and the seropositivity in young children 3–5 years old was 91.1%.

### 3.2. Associated Factors with Seropositivity against Measles

From the univariate logistic regression model, the factors that were associated with measles seropositivity were: age and having two documented doses of measles-containing vaccine. After adjustment to a multivariate logistic regression model, adolescents aged 10–18 years had a significantly lower measles seropositivity rate compared with younger children; aOR 0.29 (95% CI 0.17–0.48). Participants with an uncertain history of measles vaccination have an aOR of 0.97 (95% CI 0.63–1.49) compared with those with two documented doses of measles vaccine. This might be explained by a collinearity of the older age group and the unavailability of the vaccination booklet to review the exact date of vaccination. There was a weak inverse correlation between measles IgG titer and the time interval between the last dose of measles vaccine and the time of the blood draw (Spearman rank correlation = −0.27, *p* < 0.001).

## 4. Discussion

This cross-sectional study demonstrated a lower measles seropositivity rate among Thai children and adolescents compared with the target age-specific immunity level for measles elimination. Overall, measles seropositivity rates were 59.1% when using measles IgG levels of >200 IU/L, and 77.2% when using measles IgG levels of >120 IU/L as a cut-off criterion. Measles seropositivity rates were highest among children aged 3–5 years and then declined by age, which may be due to the waning of antibodies without natural exposure or may be due to primary vaccine failure.

According to studies in 2004 in Thailand, measles seroprotective rates were 73–76% among children aged 1–14 years and 82% among those aged 15–19 years [16]. In 2014, the seroprotective rate was highest among the age group older than 30 years (98.7%) who were naturally infected, while children 7–14 and 15–25 years old who should have received two doses of measles vaccine had measles seroprotective rates of 74.1% and 73.2%, respectively [17]. Our study also found that adolescents had a lower measles seropositivity rate compared to children, and we observed an inverse correlation of measles antibody titer by age. In the past two decades, a low rate of measles infection caused adolescents and children to lack exposure to the virus. Their measles antibody titer levels were derived entirely from vaccinations and waned as they aged because they lacked natural boosters, in contrast to adults in the past who were exposed to natural infections. This is comparable to the study in Canada, which found that measles seropositivity was 78.7% in adolescents [19]. Our study found that measles seropositivity rates in the Thai population aged 3–18 years were 82.7%, conflicting with the 2018 survey in Singapore that found that those aged 7–17 years had a 96.8% measles seropositivity rate. This may be explained by the high MMR vaccination rate of more than 96% in Singapore [20], or by a difference in sensitivity of the measles IgG assay.

Our study showed that older age and an uncertain history of measles vaccination were associated with a lower measles seropositivity rate. Published studies in China and Mexico also reported that lower measles seropositivity rates were associated with older age [21,22]. In terms of seroprevalence of MMR, the lower measles seropositivity found in this study is comparable to the previous study, which found that rubella and mumps seropositivity levels were lower among adolescents [23,24]. Our study also showed waning measles immunity over time, which was similar to a previous study of MMR seropositivity in Thailand, which reported a declination of rubella and mumps immunity, 4.2% and 12% yearly, respectively, among the fully vaccinated population [23,24].

In terms of the definition of the measles seropositivity rate, there are discrepancies between lab assays and the cut-off criteria among previous reports. Using only ELISA methods may underestimate measles antibody seroprevalence due to its low sensitivity [25]. The two-step approach of a measles IgG assay followed by PRNT in borderline/negative cases may be beneficial to validate the immunity status [26]. In a US military study, ELISA testing was first performed, finding a measles seropositivity rate of 81%. Then, samples with negative measles IgG were tested with a plaque reduction neutralization assay (PRNT). PRNT of greater than 120 mIU/mL was defined as positive for measles immunity. As a result, overall measles seropositivity increased from 81% to 96% [25]. A serosurveillance study in Canada also used a two-step approach of IgG assay followed by validation with PRNT. Using a PRNT greater than 120 mIU/mL as the cut-off for measles immunity was chosen, as this titer demonstrated protection from clinical measles [27]. Additionally, it correlated with vaccine coverage, vaccine efficacy, and the waning of antibodies over time. In our study, if we used a lower cut-off threshold of seropositive measles IgG levels of >120 U/L instead of >200 U/L, the proportion of children and adolescents who had immunity increased from 59.1% to 77.2%.

Due to the large outbreak of measles in Thailand in the past, supplementary immunization activities (SIA), including a measles and rubella vaccine campaign by the Ministry of Public Health, have been arranged since 2019 for the high-risk groups; a catch-up campaign was also arranged for children aged between 1–12 years old and adults aged between 20–40 years old in crowded settings (military conscripts, prisoners, and health personnel, etc.). However, during the COVID-19 pandemic, SIA campaigns might have slowed down from those originally planned due to many health resources, especially immunization officers, being assigned to assist with COVID-19, causing an increased immunity gap among the population. Moreover, due to the lower seropositivity rates among the adolescents in this study, the targeted adult age group of SIA campaigns may need to be targeted when they are adolescents or a third dose of MMR vaccination may be needed. However, Fiebelkorn et al. studied the immunogenicity of the third dose of the MMR vaccine in previously two-dose-vaccinated young adults, and they found that the concentration of neutralizing antibodies increased after one month post-booster compared with the baseline, but then declined almost back to baseline levels in one year. Only eight participants (1.3%) had ≥four-fold rises in measles antibody concentrations from the baseline to 1 year post vaccination [28]. Therefore, it is still controversial to determine whether a booster dose is needed and will add long term community immunity to prevent the resurgence of measles or an outbreak of measles. However, in special situations, i.e., military conscription/training where people will have to live in crowded conditions, or health care personnel with a higher risk of exposure to measles, a measles booster dose in young adults may be beneficial.

Our study has several strengths. Firstly, we have contemporary data of seropositivity surveillance of measles in Thai children and adolescents, which includes those born after the Thai Ministry of Public Health rescheduled the second dose of MMR vaccine from 4–6 years of age to 2.5 years. Secondly, we performed a PRNT assay to correlate data of measles IgG and gold standard PRNT and performed a sensitivity analysis showing seropositivity against measles in a range of 50.7–75.2% among adolescents who may need a booster dose. However, our study has limitations. Firstly, participants in this study were mostly from Bangkok and its vicinity, which may limit the generalizability of the data results. However, Thailand has a strong national immunization program with a reported measles vaccination coverage of >95% in all provinces, so we can generalize our results to other provinces in Thailand. Secondly, we did not perform PRNT in all blood samples, but randomly selected samples from three groups due to constraints in laboratory capacity. Lastly, this dataset was not able to address the issue of the interval between doses due to collinearity with the birth cohort, with the older cohort having a longer interval of 4–6 years between doses, and a longer period from the last dose to the blood draw compared with the younger birth cohort. The waning of measles IgG antibodies over time may create a bias that underestimates the protective immunity in this cross-sectional seroprevalence study. However, measles is a highly contagious virus and since global policy is moving towards measles elimination, it is crucial to have a very high seroprotective level.

## 5. Conclusions

Children younger than 5 years old met the threshold of 85% measles protective antibody. However, only half of adolescents 10–18 years of age had measles IgG above seroprotective level, making them a potentially susceptible population to measles infection in the future. The results of this study provide data in terms of humoral immunity in a population that had lower immunity than the target that was used for the measles elimination goal. Therefore, close monitoring for case surveillance should be implemented. A third dose measles booster may be necessary for those in a high-risk population or for young adults who are at risk of outbreaks, such as military conscripts.

## Figures and Tables

**Table 1:** Demographic data of 570 Thai children and adolescents in the study.

Characteristics	
Age (years)	11.7 (9.4–14.8)
Age group	
3–9 years	148 (26%)
10–18 years	422 (74%)
Sex, male	245 (43%)
Measles immunization status	
Documented medical record of 2 MCV doses	310 (54.4%)
Documented medical record of 1 MCV dose	87 (15.1%)
Medical record not available	173 (30.5%)
Age at first dose of measles vaccine (months) (*n* = 397)	9.6 (9.2–10.6)
Age at second dose of measles vaccine (years) (*n* = 310)	4.3 (3.0–5.3)
Interval from last measles vaccination to blood draw (years) (*n* = 287)	4.7 (3.1–7.1)
3–9 years (*n* = 148)	3.0 (2.3–3.5)
10–18 years (*n* = 139)	7.0 (5.0–9.4)

Data were shown as median (interquartile range) or number (percentage); MCV, measles-containing vaccine.

**Table 2:** Geometric mean titers of measles IgG and measles seropositivity rates among Thai children and adolescents.

		Measles IgG by ELISA		Measles Seropositivity Rate *		
	*n*	GMT (95%CI)	*p*-Value	*n*	% (95%CI)	*p*-Value
Total	570	281 (257–306)		337	59.1 (55.0–63.2)	
Age group			<0.001			<0.001
3–5 years	68	466 (374–580)		58	85.3 (74.6–92.7)	
6–9 years	80	361 (293–446)		58	72.5 (61.4–81.9)	
10–14 years	296	242 (213–274)		150	50.7 (44.8–56.5)	
15–18 years	126	259 (217–310)		71	56.3 (47.2–65.2)	
Sex			0.70			0.64
Male	244	287 (252–325)		147	60.2 (53.8–66.4)	
Female	326	276 (245–312)		190	58.3 (52.7–63.7)	
Measles Immunization status			0.02			0.01
Documented 2 doses	310	311 (276–349)		199	64.2 (58.6–69.5)	
Documented 1 dose	87	289 (223–351)		51	58.6 (47.6–69)	
Immunization booklet not available	173	235 (200–276)		87	50.3 (42.6–57.9)	

* Measles seropositivity rate is defined as measles IgG level of >200 IU/L, measured by ELISA method (EUROIMMUN).

**Table 3:** Sensitivity analysis of measles seropositivity rate among Thai children and adolescents.

			Proportion of Measles Seropositivity with Different Measles IgG Cutoffs		
Age (Years)	*n*	IgG: GMT (95%CI)	>120 IU/L	>200 IU/L	>275 IU/L
3–5	68	466 (374–580)	91.1 (81.8–96.7)	85.3 (74.6–92.7)	72.1 (59.9–82.3)
6–9	80	361 (293–446)	88.8 (79.7–94.7)	72.5 (61.4–81.9)	58.8 (47.2–69.6)
10–14	296	242 (213–274)	71.3 (65.8–76.4)	50.7 (44.8–56.5)	38.9 (33.3–44.7)
15–18	126	259 (217–310)	75.2 (67.8–83.3)	56.3 (47.2–65.2)	42.1 (33.3–51.2)
Overall	570	281 (257–306)	77.2 (73.5–80.6)	59.1 (54.9–63.2)	46.3 (42.2–50.5)

Note: The Thai national program recommended two doses of measles-containing vaccine since 1997; children aged 3–5 years received the second dose of measles vaccine at the age of 2.5 years, while older children received the second dose of measles vaccine at 4–6 years of age.

## Data Availability

The data supporting this study’s findings are available from the corresponding author upon reasonable request.
